# Strategies and Educational Approaches to Colonoscopy Training: A Scoping Review

**DOI:** 10.1111/tct.70371

**Published:** 2026-03-04

**Authors:** Bernard K. Le, Jonathan S. Y. Hong, Daniel Lee

**Affiliations:** ^1^ Institute of Academic Surgery Royal Prince Alfred Hospital Sydney New South Wales Australia; ^2^ Central Clinical School, Faculty of Medicine and Health University of Sydney Sydney New South Wales Australia; ^3^ Department of General Surgery Canterbury Hospital Campsie New South Wales Australia

**Keywords:** colonoscopy, endoscopy, general surgery, simulation, surgical education

## Abstract

Learning colonoscopy is challenging for inexperienced endoscopists, with competence typically achieved through observation, verbal instruction and hands‐on clinical training.

**Objective:**

Various non‐clinical teaching models have been developed and are used globally. This scoping review identifies these methods and evaluates their learning outcomes.

**Design:**

Following the PRISMA‐ScR framework, MEDLINE and Embase databases were searched for English‐language articles published from 2012 onward. Two reviewers screened eligible studies, resulting in a final selection of relevant full‐text articles. The review addressed (1) which teaching methods/tools have been tested for endoscopy training and (2) which methods yield better colonoscopy performance outcomes.

**Results:**

From 217 articles, 31 met inclusion criteria. The most common teaching methods were virtual reality (VR) models and physical models, both within and across studies. Other studies included box models, animal models, real‐time feedback devices, traditional/verbal instruction models, didactic models and two‐person colonoscopy models. Across all studies, participants ranged widely in experience from medical students to experienced specialists, with the effectiveness of training modalities closely tied to the participants' baseline experience.

**Conclusion:**

Despite the variety of colonoscopy teaching methods, there is limited comparative research on their effectiveness both alone and when integrated with the traditional/verbal learning model. Prior experience appears to be a clear predictor of any model's potential effectiveness. As such, it appears important to tailor any colonoscopy training method to a learner's experience level to optimise skill acquisition and clinical readiness. Further research would benefit from a direct comparison of all teaching methods across a range of user skill sets.

## 1 Introduction

Endoscopy has indications in assessing abnormalities in imaging studies, investigating gastrointestinal bleeding, iron‐deficiency anaemia and inflammatory bowel disease and preventing colorectal cancer (CRC) progression [1] through detection and removal of neoplastic lesions. In the context of CRC, such early‐stage detection is associated with very high cure rates, underscoring the importance of colonoscopy procedures [2, 3].

Historically, colonoscopy training has been a non‐standardised endeavour carried out in an apprenticeship‐like fashion, completely procedural and live in the clinical setting [4]. Although there are some benefits to this procedural setting model, such as situational realism and live feedback, it also comes with limitations. These limitations include time management challenges for the multiple stakeholders present, the promotion of a ‘trial and error’ culture, insufficient time for long‐form supervisor feedback, no time for self‐reflection and, most importantly, the potential for injury to the patient [5].

Historically, colonoscopy training has been a non‐standardised endeavour carried out in an apprenticeship‐like fashion.

There are guidelines available to assist educators and trainees throughout the learning process [6, 7]; however, they remain recommendations that are often not widely adopted as standardised methods or curriculum for optimal colonoscopy training.

As is apparent in all pursuits of skill acquisition, the pace of learning can vary greatly among trainees [8, 9]. Slow learners struggle to achieve the intended objectives after multiple attempts. Faster learners find themselves stalled in progression waiting for the end of a fixed‐length training program [10].

Recognising the limitations of this traditional/verbal model, colonoscopy instructors have sought innovative methods to enhance endoscopic training of novices. The growing importance of patient safety has brought simulation‐based training to the forefront [5]. However, when it comes to simulation training, there is no consensus on the management of training programs, type of equipment to use, duration for training or need for supervision [10].

The growing importance of patient safety has brought simulation‐based training to the forefront.

Various colonoscopy learning and teaching models have been produced and are currently used worldwide. Virtual reality (VR) models use computer‐generated simulations to replicate the colonoscopy environment, allowing trainees to practise in an immersive, risk‐free setting. Physical models are anatomical replicas of the colon and enable hands‐on practice of scope manipulation. Box models are simplified physical trainers, typically consisting of a box with internal pathways that mimic the colon structure and are designed to teach basic navigation and hand–eye coordination. In vivo (animal) models allow procedures on live or cadaveric animals, offering realistic tissue handling and physiological responses. Real‐time feedback devices, including VR simulators or colonoscopy tools, provide immediate performance metrics, including scope position, force applied and completion time, and help trainees adjust techniques during practice. Traditional/verbal models rely on direct observation and verbal instruction from experienced endoscopists during live procedures, emphasising mentorship and experiential learning. Similarly, the two‐person colonoscopy technique involves one operator controlling the insertion and withdrawal of the scope while another manages torque and angulation, reducing physical strain during training. Lastly, a less practical alternative is the didactic model, which focuses on teaching through lectures and demonstrations to provide foundational understanding before practical application.

The purpose of this review is to gain an understanding of the teaching tools and methods currently available. A scoping review was conducted to broadly examine the extent, range and nature of colonoscopy training and to produce a clear and concise summary of teaching tools available. Our specific research questions included the following:

- What teaching methods/tools are available to endoscopy trainers that have been analytically tried and tested?
- What teaching methods have yielded more efficacious outcomes in regard to colonoscopy performance, inclusive of caecal intubation success/rate, withdrawal and tip control?

## 2 Methods

This study was carried out using the Preferred Reporting Items for Systematic Reviews and Meta‐Analysis Extension for Scoping Reviews (PRISMA‐ScR) [11] and the methodological framework laid out by Arksey and O'Malley [12].

### 2.1 Eligibility Criteria

To be eligible for inclusion, studies were required to report on colonoscopy training with a clear focus on learning outcomes inclusive of technical skill and patient outcomes. To ensure consistent assessment across studies, the teaching method had to be explicitly described and considered reproducible. Editorials, abstracts only, commentaries and studies lacking a defined training method were excluded.

Studies that focused on a specific outcome such as caecal intubation, withdrawal or tip control as surrogate markers of completion were included. Advanced colonoscopy techniques such as polypectomy‐specific teaching were excluded, except in instances where they were used alongside alternative models that trained general colonoscopy.

All levels of learners were included, inclusive of medical students, residents and qualified clinicians and doctors. Novice was defined as a participant who had no prior experience with endoscopy or very limited exposure, with the included studies limiting endoscopy experience to at most 20 endoscopies performed in clinic or simulation. The review was limited to studies published between 2012 and 2024 to ensure sufficient studies were assessed to provide a valuable assessment across techniques while maintaining clinical relevance to current training practices. Only studies published in English were included due to limitations in reviewer language proficiency.

### 2.2 Search Strategy

The initial search used MEDLINE and Embase databases. In both databases, the following keywords were searched: Colonoscopy AND (Education, Medical[MeSH] OR Education, Medical, Graduate[MeSH] OR Teaching[MeSH] OR ‘teaching methods’[Title/Abstract] OR ‘colonoscopy training’[Title/Abstract]).

This search strategy was employed twice, on 1 September 2021 and again on 7 November 2024. Given the time elapsed between the initial protocol development and the manuscript submission, the 7 November 2024 search was conducted using identical terms and databases to identify studies published since the original search. Both searches were combined and de‐duplicated prior to eligibility screening. A protocol was not registered for this review.

### 2.3 Identifying Relevant Studies

The identified studies were reviewed independently by two reviewers (BL and DL). Duplicates were removed, and the titles and abstracts were reviewed, retaining only the studies relevant to this study's focus. The full publications of the remaining articles were acquired for independent review by both reviewers.

Reviewers manually screened all studies based on

- Studies reporting on colonoscopy training specifically
- Learning outcomes before and after the implemented method
- Models, methods and technologies that are readily accessible commercially or reproducible

Instances of reviewer disagreement on article relevance to the eligibility requirements were resolved by joint reassessment of the full text for eligibility used for inclusion, or instances of initial disagreement were individually discussed and articles were included or excluded accordingly. Studies upon which reviewers could not reach a consensus were excluded.

In studies with multiple teaching methods, the methods were treated as distinct entities for broader comparison, rather than as varying sub‐categories of the same method (for instance, physical simulator model vs. magnetic positioning device, rather than physical simulator model plus magnetic positioning device). This approach was implemented to determine the frequency of use relative to other modes and to evaluate each teaching method individually.

During the scoping process, articles were broken down and processed into each article's research focus, participant population, methodology and results. This processed version of articles was shared by both reviewers and used to produce this article and its figures and tables.

## 3 Results

**FIGURE A1:**
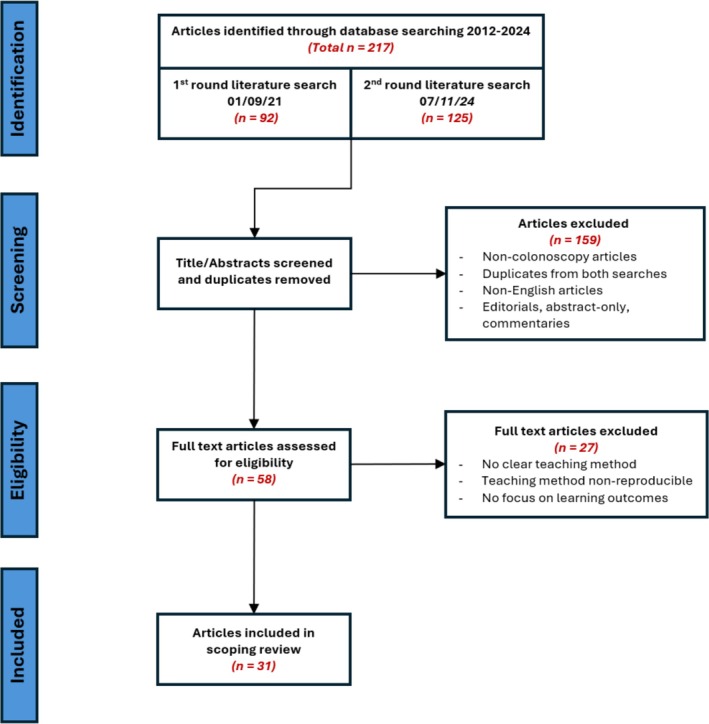
Flow diagram of study selection.

A total of 217 articles were identified from the primary MEDLINE and Embase searches (Figure A1), with 92 articles identified in the September 2021 search and 125 in the November 2024 search. Following review, the updated search identified an additional 16 studies meeting the inclusion criteria and included for the final appraisal.

After excluding duplicates and inappropriate articles, 58 articles remained. From these, 27 articles were excluded following complete review, as they did not fit the eligibility criteria, for example, due to an emphasis on lesion detection rates, a focus on teaching method design but not on application or insufficient detail of the teaching method. Thirty‐one articles were included in the final analysis for this scoping review.

Across articles, the following teaching modalities were assessed: VR, physical model, box model, animal model, real‐time feedback devices, traditional/verbal method, didactic model and two‐person colonoscopy.

A summary of the studies included is shown in Table A1.

Table A2 shows the frequency of studies that assessed each method and the frequency of method instances across all studies. In both cases, the VR and physical models were the most frequently assessed, studied in 52% and 42% of all studies, respectively, and were representative of 33.3% and 27% of all instances.

VR simulators demonstrated overall positive outcomes for colonoscopy training. Three VR‐based studies evaluated the effectiveness of VR as a tool for assessing learning strategies. Key findings indicated that structured training outperformed self‐directed learning, and progressive learning led to better outcomes than a standard structured curriculum. In addition, a correlation was found between VR performance and video game proficiency. One study reported that there was a discrepancy in skill transfer competency in caecal intubation via VR and that it did not correlate with performance during hands‐on clinical practice [15, 17, 18, 27].

In one study, VR was compared with a commercially available physical model, and although both showed significant improvement, utilising VR was found to have significantly better performance [21]. A VR simulator training program (50–100 sessions) showed marked improvements in the time to caecal intubation rate (9.5 vs. 2.2 min, *p* = 0.002) and colonic insertion depth (29.4 vs. 63.7 cm, *p* < 0.001) in a cohort of 18 trainee doctors without previous colonoscopy experience [19]. Lastly, one study [16] showed that the VR alone, with cross‐training to a physical model, significantly improved outcomes compared to the physical model alone.

Physical models were the second most prevalent training paradigm identified in the literature of endoscopic simulation. These models [15] drove accelerated task completion in the majority of included articles, although two studies did not directly evaluate the physical models as a learning tool [27, 29]. One study showed that a physical model exclusively (without cross‐training on a VR model) did not improve performance in scope navigation, advancement or lumen view [16]. Another study [31] found that simulated training on a low‐cost physical model increased rates of colonoscopy completion and scope withdrawal technique in 12 interns lacking previous colonoscopy exposure. Furthermore, the mean procedure time decreased (24 min 46 s to 20 min 54 s [*p* = 0.03]), incomplete colonoscopy rate decreased (100% to 33.3% [*p* = 0.042]) and duration of loss of lumen view decreased (75% to 8.3% [*p* = 0.023]).

The box model was the third most frequently assessed methodology, assessed across 16% of all studies and 10.4% of instances. Within this category, models were highly varied and typically designed to target specific colonoscopy techniques or skills. For example, bench‐top models [18, 25] and computerised tip‐control devices [18, 37, 39] were used for tip manipulation training. Comparatively, inanimate plastic colon tube models [37, 38] were used to teach scope torque techniques. One tip‐control‐specific box model found that box model training reduced performance differences between novices and experienced colonoscopists and significantly reduced procedure times for novices compared other their initial baseline performance.

Animal simulators were described in four studies, with evaluation of both in vivo and ex vivo models [23, 28, 37, 38]. Whereas two of four animal models [37, 38] were utilised for endoscopic submucosal dissection (ESD) training, this was done alongside alternative models that trained general colonoscopy. As such, these studies were included within this review. In vivo animal models with blood flow, such as porcine models, have been successfully used for teaching ESD training [43]. One study [23] found that a 7‐month training regimen on an ex vivo model yielded a high success rate in mid‐career general internists, even outperforming experienced colonoscopists in some areas. Similarly, a review [28] found that animal models offered benefits for training in EMR (endoscopic mucosal resection) and ESD procedures, although acknowledged that training in other skills such as scope insertion were better addressed by physical or VR models.

Real‐time feedback devices, including colonoscopy‐mirroring tablet applications [42], magnetic positioning devices [28, 32] and physical models with built‐in computerised feedback scoring [36], were assessed in four studies. One study found improved performance when using these devices in live patients [28]. Another study used computerised feedback score system coupled to a physical model, showing that participants provided with feedback scores outperformed those who did not receive feedback [36]. A colonoscopy‐mirroring application in the form of a trainer‐controlled tablet [42] found that participants missed fewer lesions and experienced lower levels of stress in this teaching environment due to improved communication facilitated by the tablet. This same approach used in polyp detection [42] found that fellows missed fewer polyps using the tablet versus traditional precepting (4.2% to 12.5% detected [*p* = 0.04]), again reporting that trainees experienced improved communication and reduced stress.

The tradition/verbal method was evaluated in three studies. One article [40] found this approach beneficial for learners when cognitive load was not excessive and when applied using the four teaching activities with positive effect (schema teaching, taking over, checking understanding and setting expectations), resulting in a positive correlation of moderate strength (Spearman's rho 0.44) with measured germane load or the cognitive effort dedicated to processing and understanding materials in a meaningful way. Feedback before and after procedures controlled cognitive burden and aided in the learning process, whereas inordinate application of teaching during the procedure had detrimental effects to the learning. Other studies combined the traditional/verbal teaching with multi‐modal programs [4, 44].

Didactic teaching methods [4, 25] were only used in conjunction with other teaching methods, such that these results are inseparable from the efficacy of other modes of teaching. Similarly, only one study assessed the two‐person colonoscopy method [41], finding no difference in completion rates (*p* = 0.382), mean detection of polyps (*p* = 0.434) or intubation lengths (*p* = 0.939), as compared to single‐person colonoscopy operation in training.

## 4 Discussion

### 4.1 Summary of Evidence

This review mapped eight distinct training modalities in colonoscopy and highlighted their roles within contemporary education frameworks. VR simulators and physical models remain widely used in the current practice and were effective for novice learners, particularly in the early phases of skill acquisition. These findings align with the growing emphasis on simulation‐based education as a structured, learner‐focused complement to traditional apprenticeship models, enabling deliberate practice prior to patient contact.

This review mapped eight distinct training modalities in colonoscopy and highlighted their roles within contemporary education frameworks.

Importantly, participant experience varied widely across studies, from medical students and early interns with no prior procedures to PGY1–PGY5 residents, novice endoscopists (< 10–20 colonoscopies), mid‐career internists, experienced consultants (> 350 colonoscopies), gastroenterologists, surgeons and trainers. Novices (< 20 colonoscopies) showed the most robust improvements within structured curricula incorporating VR and physical models [19, 20, 25]. For this cohort, scaffolded instructional designs proved particularly effective in accelerating skill acquisition [14, 17, 18], in independent practice [24] and in feedback‐enhanced training [36], which further strengthened performance. In contrast, for more experienced participants, gains were attenuated. For example, training with a real‐time feedback device standardised performance between novices and experienced colonoscopists and improved novice outcomes from baseline. However, experienced colonoscopists did not improve. This suggests a plateau for certain tools that emphasise foundational skills (e.g., scope manoeuvring). Indeed, Mitra et al. reported performance plateaus after repetitive practice, indicating diminishing returns without progressive task complexity [37].

Novices (< 20 colonoscopies), showed the most robust improvements within structured curricula incorporating VR and physical models.

Experienced practitioners outperformed less experienced cohorts on complex tasks with targeted interventions; however, real‐time feedback and intensive programs helped narrow performance gaps. Most notably, in one study, a 7‐month training regimen standardised colonoscopy performances between general internists and gastroenterology attendings, reinforcing the importance of repeated practice for colonoscopy success rates. However, endoscopist level alone should not be the sole factor considered in assessing training modalities, as the background level of the individual may not reflect their prior endoscopy exposure. It is also notable that many studies did not adequately assess the impact of feedback. This is despite its centrality to surgical skill acquisition, as evidenced by the numerous structured tools (DOPS, global rating scales [GRS], ACE and GiECAT) that provide standardised evaluation across domains (e.g., scope handling, mucosal visualisation, patient safety and communication) [23, 32, 42].

### 4.2 Limitations/Strengths

Limitations in this study comprise the potential exclusion of unpublished data and grey literature through this study's methodology of literature scoping from scientific literature alone. Consequently, valuable insights and data that reside outside the conventional publishing frameworks may be overlooked, leading to an incomplete representation of the research landscape.

The evidence is also constrained by heterogeneity in participant experience, outcome measures, study designs and the overrepresentation of VR and physical models. Several studies did not isolate the contribution of feedback or differentiate effects across specific skill domains. Given the variability in prior exposure, generalisations for instruction based on PGY level are limited.

Most importantly, the absence of direct comparisons across all methods is a limitation. Without comparing methods between individuals of the same baseline skill set, it is difficult to determine the most effective training method.

## 5 Conclusion

Simulation‐centred training, including VR and physical models, supported early colonoscopy skill acquisition, while progressive complexity and robust feedback were essential for sustaining improvement and closing performance gaps.

Simulation‐centred training, including VR and physical models, supported early colonoscopy skill acquisition.

The findings of this study support that adaptive, competency‐based curricula that front‐load VR and structured simulation for novices accelerate early skill acquisition. These simulation‐based approaches also reduce cognitive load during initial patient encounters. Consideration should be given to embedding progressive complexity into teaching instruction paradigms (e.g., graded tasks, scenario variability and complications management) to minimise plateauing in skill acquisition and to generate teaching tools that are equally valuable across a range of experience levels. Aligning modality selection with baseline exposure rather than training level alone would ensure optimal skill acquisition. Feedback infrastructures such as DOPS, ACE, GiECAT and GRS should also remain standard across teaching tools, and all tools can be integrated with more traditional/verbal approaches to further promote learning.

Future research into these learning models should attempt to ascertain and directly compare the efficacies of each model and to arrive at comparable outcomes across all used teaching methods. Extension of this scoping review should explore training of advanced colonoscopy techniques, such as advanced polypectomy.

Future research into these learning models should attempt to ascertain and directly compare the efficacies of each model.

## Author Contributions

**Bernard K. Le:** conceptualization, methodology, investigation, data curation, writing – original draft, writing – review and editing, visualization. **Jonathan S. Y. Hong:** validation, writing – original draft, writing – review and editing, visualization, supervision, project administration. **Daniel Lee:** conceptualization, methodology, data curation, writing – original draft, writing – review and editing, visualization, supervision, project administration.

## Funding

The authors have nothing to report.

## Conflicts of Interest

The authors declare no conflicts of interest.

## Data Availability

Data sharing is not applicable to this article as no datasets were generated or analysed during the current study.

## Appendix A

**TABLE A1:** Characteristics of the 31 included articles organised by teaching method.

Simulation			
Author	Research focus	Participants	Findings
Virtual reality (VR) model			
Aljamal et al. (2018) [ 13 ]	Developing a simulator‐based curriculum in teaching doctors	66 residents 30 PGY1 12 PGY2 8 PGY3 9 PGY4 7 PGY5	Prior experience with the computer‐based endoscopy simulator strongly correlated with successful task completion. Voluntary hands‐on teaching with the simulator allowed for 96% of the cohort to achieve task completion.
Cassidy et al. (2022) [ 14 ]	Evaluating skill transfer from VR endoscopy training to live porcine endoscopic performance	22 novice residents (< 10 colonoscopy experience)	Reduced time spent completing upper and lower endoscopy tasks in live animal model. Proficiency‐based curriculum was found to be less time consuming compared to repetition‐based curriculum.
Elvevi et al. (2012) [ 15 ]	Examine the usefulness of using a computer‐based simulator as an objective measure during traditional hands‐on colonoscopy training	12 colonoscopy trainees (fully trained in upper GI endoscopy) 15 experts (control group)	Computer‐based simulators were not useful in evaluating competence during hands‐on training in colonoscopy.
Gomez et al. (2015) [ 16 ]	Compare simulators and their respective roles in transferring skills to the clinical environment	27 surgical (postgraduate first‐year residents)	GI mentor (VR model) and combined simulator groups improved GAGES total score. The Kyoto (physical model) alone did not.
Grover et al. (2015) [ 17 ]	Compare simulation‐based structured comprehensive curriculum (SCC) to self‐regulated learning (SRL) for colonoscopy competency	33 novice endoscopists (< 20 colonoscopy experience) Gastroenterologists General surgeons Internal medicine	Self‐regulated learning did not change competency. SCC showed superior technical and non‐technical performance in both the first and second clinical colonoscopies. Better knowledge, colonoscopy‐specific performance, communication and global ratings in the integrated scenario.
Grover et al. (2017) [ 18 ]	Simulation‐based training through SCC vs. progressive learning–based curriculum (PLC)	37 novice endoscopists 18 PLC 19 SCC	The PLC group outperformed the SCC group with superior technical, communication and overall performance in both colonoscopies. No difference in endoscopic knowledge between PLC and SCC.
Koch et al. (2015) [ 19 ]	Use of a VR endoscopy simulator result in improved clinical performance	18 trainees (without endoscopy experience)	Significantly improved caecal intubation time, insertion depth and intubation rate on the simulator. Corresponding performance gains during patient‐based colonoscopy.
McIntosh et al. (2014) [ 20 ]	Virtual reality simulator training translating to improved patient‐based colonoscopy performance	18 residents PGY2–PGY4 (no previous colonoscopy experience)	Better mean depth of unassisted insertion in the simulator‐trained group vs. the control group. No significant difference in insertion time or patient discomfort.
Mu et al. (2024) [ 21 ]	VR simulator vs. physical model simulators	40 surgical residents	Both the VR and physical model groups significantly improved compared to baseline. VR simulators performed significantly better than the physical model.
Ortolani et al. (2015) [ 22 ]	Can junior‐level residents learn to conduct competent and safe screening colonoscopy through a structured curriculum	5 postgraduate second‐year residents (supervised by surgical endoscopists)	Curriculum incorporating a dedicated endoscopy rotation, extensive simulation training and consistent faculty‐led teaching. Resulted in junior surgical residents performing safe and competent screening colonoscopy.
Shah‐Ghassemzadeh et al. (2017) [ 23 ]	Use of intensive training program to train mid‐career general internist in high‐quality colonoscopy	A single general internist (performing 989 colonoscopies)	General internist attained high success rates in quality measures.
Telem et al. (2014) [ 24 ]	Effectiveness of independent virtual endoscopic training in accelerating the acquisition of endoscopic skill	9 novice interns (experimental group) 3 attending physicians (control group)	Independent training significantly improved endoscopic skills in novice surgical interns as demonstrated in swine models. Study identified parameters that could indicate clinical readiness.
Walsh et al. (2020) [ 25 ]	Efficacy of simulation‐based curriculum with dedicated non‐technical skills (NTS) training in improving clinical performance	42 novice endoscopists (< 20 colonoscopy experience)	The NTS group achieved higher JAG DOPS scores than controls in both clinical colonoscopies. Case difficulty was comparable initially but significantly lower in the second procedure for the NTS group. Knowledge scores were comparable, whereas communication and colonoscopy‐specific skills were significantly higher in the NTS group.
Williams et al. (2015) [ 26 ]	Effect of endoscopy simulation curriculum on quality of performance by general surgery residents	598 colonoscopies by gastroenterology residents 220 colonoscopies by general surgery residents	Adenoma detection and caecal intubation rates were similar between groups. Gastroenterologists performed more biopsies per colonoscopy, whereas general surgeons biopsied more adenomas per colonoscopy.
Willis et al. (2014) [ 27 ]	Correlation between VR or physical model training with video game efficacy	20 laparoscopic novices (first‐ and second‐year medical students)	No significant correlation with video games and physical model. Video games Level 12 correlated with VR. No correlation between physical model and VR.
Yoshida et al. (2014) [ 28 ]	Reviewing training methods for colonoscopic examination and treatments	Review of literature	Enabled practice around patient discomfort but was less effective for spatial orientation, haptics and loop management. Its effectiveness was limited to the early phase of the learning curve.
Physical model			
Aljamal et al. (2019) [ 29 ]	Assessing the use of participant confidence boosting and motivation before undergoing a task	40 participants 30 undergrad students 10 medical students	Performance expectations were influenced by pre‐induced conceptions. Participants given a low predicted success rate performed better, likely from increased focus, whereas those given a high prediction were more confident but less effective.
Aljamal et al. (2019) [ 30 ]	Colonoscopy technique training via physical model simulation	40 trainees General surgery interns Research fellows Medical students	Caecal intubation rose from 10% pre‐training to 100% post‐training, with 96% still reaching the caecum at 2‐week retention testing. Participants reported superior haptic feedback compared to the computer simulator.
Buscaglia et al. (2015) [ 31 ]	Improved responsiveness using a simulated physical model	12 interns (colonoscopy novices)	Simulated colonoscopy training improved surgical intern performance, reducing procedure time, increasing completion rates and promoting appropriate scope withdrawal.
Gomez et al. (2015) [ 16 ]	Compare simulators and their respective roles in transferring skills to the clinical environment	27 surgical first‐year residents	GI mentor (VR model) and combined simulator groups improved GAGES total score. The Kyoto (physical model) alone did not.
Mu et al. (2024) [ 21 ]	VR simulator vs. physical model simulators	40 surgical residents	Both the VR and physical model groups significantly improved compared to baseline. VR simulators performed significantly better than the physical model.
Nerup et al. (2015) [ 32 ]	Validity of using magnetic endoscopic imaging for assessment and training of endoscopists	10 experienced endoscopy consultants (> 350 colonoscopy experience) 11 trainees (< 2 colonoscopy experience)	Consultants outperformed trainees across all difficulty levels, with significant performance differences. Magnetic imaging enabled unbiased performance assessment, real‐time feedback and enhanced training progression.
Plooy et al. (2016) [ 33 ]	Unsupervised practice of a brief structured program on a physical model colonoscopy simulator in novice endoscopists	32 first‐year medical students (no endoscopic experience) Compared to an experienced colonoscopists	Post‐program participants were better prepared for supervised live cases, demonstrating improved completion rates, reduced time to caecum and lower peak force applied. Post‐test trainees outperformed untrained novices, whereas experienced colonoscopists maintained superior performance overall.
Ritter et al. (2018) [ 34 ]	Feasibility of utilising a novel physical simulator with curriculum to prepare trainees for manual skills exam	11 experienced endoscopists (3 colorectal surgeons, 4 gastroenterologists, 4 GI surgeons) 17 novice residents (10 PGY1, 7 PGY2)	Three trainees passed the pre‐training FES. After training, 71% reached initial and 43% full proficiency across all five ETS tasks, with similar FES manual skill gains in PGY1 and PGY2 trainees.
Ritter et al. (2019) [ 35 ]	Designing a low‐cost, high‐fidelity endoscopic skills model to help surgical trainees pass the Fundamentals of Endoscopic Surgery (FES) testing	Surgery residents PGY1–PGY5	No formal evaluation or validation of the model was conducted. Anecdotal feedback was positive regarding realism, accessibility, affordability, confidence and curriculum integration. This low‐cost model addresses the need for a high‐fidelity, easily implemented training tool across surgical programs.
Vilmann et al. (2018) [ 36 ]	Use of a computer‐generated feedback score in improving performance on a physical simulation model	44 participants (interns/JMOs)	The feedback group outperformed controls with faster times, improved motion efficiency and higher scores. They practised the easy case more often, reaching the caecum 3.2× more frequently, whereas controls focused only on caecal intubation as success.
Waschke et al. (2016) [ 4 ]	An effective standardised training program for trainers of colonoscopy	Program caters to 3–6 endoscopy trainers with previous endoscopy experience	Effective standardised colonoscopy training requires competent endoscopy trainers. Implementation of the program has substantially improved colonoscopy quality outcomes across the United Kingdom.
Willis et al. (2014) [ 27 ]	Correlation between VR or physical model training with video game efficacy	20 laparoscopic novices (first‐ and second‐year medical students)	No significant correlation with video games and physical model. Video games Level 12 correlated with VR. No correlation between physical model and VR.
Yoshida et al. (2014) [ 28 ]	Reviewing training methods for colonoscopic examination and treatments	Review of literature	The physical model simulator allows practice in tip control and proprioceptive manoeuvring but lacks evidence of efficacy. It remains an affordable training option.
Box model			
Grover et al. (2017) [ 18 ]	Simulation‐based training through SCC vs. PLC	37 novice endoscopists 18 PLC 19 SCC	A bench‐top box colonoscopy model was used. The PLC group outperformed the SCC group with superior technical, communication and overall performance in both colonoscopies. No difference was observed in endoscopic knowledge between groups.
Mitra et al. (2024) [ 37 ]	Using inanimate and ex vivo animal teaching models to learn and repeatedly practise new skills	8–17 MDs trained on Modules 1–3 5 MDs trained on Modules 4 and 5	An inanimate plastic colon tube model was used. Completion times for lesion location decreased and plateaued after 6–8 sessions (Module 1). Using ex vivo porcine bowel, trainees mastered wall injection differentiation with two reported increased confidence and later performed ESD successfully (Modules 2 and 5).
Mitra et al. (2024) [ 38 ]	Assessing preliminary performance outcomes in MDs using inanimate and ex vivo animal teaching models	7–8 MDs	An inanimate plastic colon tube model was used. MD participants demonstrated improved submucosal injection (Module 1), scope manoeuvrability (Module 2), higher ESD success on ex vivo tissue and increased overall confidence (Module 4).
Riek et al. (2017) [ 39 ]	Development of a training device for colonoscopic tip control and assessment of its efficacy	Convenience sample 13 experienced colonoscopists 10 gastroenterologists 2 general medicine MDs 1 nurse endoscopist 16 novices (first‐ and second‐year medical students)	A computerised tip‐control training device was used. Colonoscopists performed faster and with less variability than novices, who improved speed and consistency with training. Post‐training, novice tip control matched experienced colonoscopists, confirming the device's effectiveness for fundamental skill development.
Walsh et al. (2020) [ 25 ]	Efficacy of simulation‐based curriculum with dedicated NTS training in improving clinical performance	42 novice endoscopists (< 20 colonoscopy experience)	A bench‐top simulator model was used. The NTS group achieved higher JAG DOPS scores than controls in both colonoscopies. Case difficulty was similar initially but lower in the second for the NTS group, indicating improved adaptability. Knowledge scores were similar, whereas communication and technical skills remained higher in the NTS group.
Animal model			
Mitra et al. (2024) [ 37 ]	Using inanimate and ex vivo animal teaching models to learn and repeatedly practise new skills	8–17 MDs trained on Modules 1–3 5 MDs trained on Modules 4 and 5	An ex vivo porcine material was used. Completion times for lesion location decreased and plateaued after 6–8 sessions (Module 1). Using ex vivo porcine bowel, trainees mastered wall injection differentiation with two reported increased confidence and later performed ESD successfully (Modules 2 and 5).
Mitra et al. (2024) [ 38 ]	Assessing preliminary performance outcomes in MDs using inanimate and ex vivo animal teaching models	7–8 MDs	An ex vivo bovine or porcine material was used. MD participants demonstrated improved submucosal injection (Module 1), scope manoeuvrability (Module 2), higher ESD success on ex vivo tissue and increased overall confidence (Module 4).
Shah‐Ghassemzadeh et al. (2017) [ 23 ]	Use of intensive training program to train mid‐career general internist in high‐quality colonoscopy	A single general internist (performing 989 colonoscopies)	An ex vivo bovine or porcine material was used. General internist attained high success rates in quality measures.
Yoshida et al. (2014) [ 28 ]	Reviewing training methods for colonoscopic examination and treatments	Review of literature	Live animal and ex vivo bovine or porcine materials were used. Currently, the only platform for ESD practice, enabling training in manipulation control and snaring under difficult conditions. Evidence for its efficacy remains limited.

Apprenticeship			
Author	Research focus	Participants	Findings
Traditional/verbal method			
Sewell et al. (2019) [ 40 ]	Germane vs. extraneous cognitive load in response to teaching in the procedural setting	8 gastroenterology fellows (supervised by 10 attending physicians)	Clinical teaching during colonoscopy enhanced learning by increasing germane load and reducing intrinsic load. Excessive guidance reduced autonomy and increased cognitive load; balanced teaching with pre‐ and post‐procedure discussion optimised learning.
Shah‐Ghassemzadeh et al. (2017) [ 23 ]	Use of intensive training program to train mid‐career general internist in high‐quality colonoscopy	A single general internist (performing 989 colonoscopies)	General internist attained high success rates in quality measures.
Waschke et al. (2016) [ 4 ]	An effective standardised training program for trainers of colonoscopy	Program caters to 3–6 endoscopy trainers with previous endoscopy experience	Effective standardised colonoscopy training requires competent endoscopy trainers. Implementation of the program has substantially improved colonoscopy quality outcomes across the United Kingdom.
Two‐person colonoscopy			
Wu et al. (2021) [ 41 ]	Evaluate the effectiveness of a two‐person colonoscopy technique in training general surgery residents for colonoscopy procedures, compared with the single‐operator method	2 third‐year residents (previous colonoscopic training via a physical model) 5 staff members	Trainees achieved acceptable outcomes over an 81–97 case learning curve under a two‐person non‐sedation colonoscopy technique. An approach with potential as a transition to single‐operator colonoscopy.

Cognitive			
Author	Research focus	Participants	Findings
Didactic model			
Walsh et al. (2020) [ 25 ]	Efficacy of simulation‐based curriculum with dedicated NTS training in improving clinical performance	42 novice endoscopists (< 20 colonoscopy experience)	The NTS curriculum was used. The NTS group achieved higher JAG DOPS scores than controls in both colonoscopies. Case difficulty was similar initially but lower in the second for the NTS group, indicating improved adaptability. Knowledge scores were similar, whereas communication and technical skills remained higher in the NTS group.
Waschke et al. (2016) [ 4 ]	An effective standardised training program for trainers of colonoscopy	Program caters to 3–6 endoscopy trainers with previous endoscopy experience	The seminar and classroom‐based curriculum was used. Effective standardised colonoscopy training requires competent endoscopy trainers. Implementation of the program has substantially improved colonoscopy quality outcomes across the United Kingdom.

Feedback			
Author	Research focus	Participants	Findings
Real‐time feedback devices			
Laborde et al. (2017) [ 42 ]	Use of ScopeVUe‐mirroring tablet application for real‐time annotation of colonoscopy view in procedural training	15 trainees 6 novices (no experience) 9 GI fellows (> 50 colonoscopy experience)	Fellows missed fewer polyps using the tablet than with traditional precepting, whereas novices showed no difference. Fellows outperformed novices across methods, and both attendings and trainees reported reduced stress and improved communication with tablet use.
Nerup et al. (2015) [ 32 ]	Validity of using magnetic endoscopic imaging for assessment and training of endoscopists	21 participants 10 experienced endoscopy consultants (> 350 colonoscopy experience) 11 trainees (< 2 colonoscopy experience)	Consultants outperformed trainees across all difficulty levels, with significant performance differences. Magnetic imaging enabled unbiased performance assessment, real‐time feedback and enhanced training progression.
Vilmann et al. (2018) [ 36 ]	Use of a computer‐generated feedback score in improving performance on a physical simulation model	44 participants (interns/JMOs)	The feedback group outperformed controls with faster times, improved motion efficiency and higher scores. They practised the easy case more often, reaching the caecum 3.2× more frequently, whereas controls focused only on caecal intubation as success.
Yoshida et al. (2014) [ 28 ]	Reviewing training methods for colonoscopic examination and treatments	Review of literature	A magnetic positioning device was used. Significantly improved colonoscopy performance, enabling faster intubation times and higher completion rates.
Waschke et al. (2016) [ 4 ]	An effective standardised training program for trainers of colonoscopy	Program caters to 3–6 endoscopy trainers with previous endoscopy experience	The seminar and classroom‐based curriculum was used. Effective standardised colonoscopy training requires competent endoscopy trainers. Implementation of the program has substantially improved colonoscopy quality outcomes across the United Kingdom.

*Note:* Summary of the 31 included articles in the scoping review. Entries are partitioned into groups of teaching methods highlighted in the articles. Articles can be listed more than once if referencing multiple modes of teaching.

Abbreviations: ESD – endoscopic submucosal dissection, GAGES – Global Assessment of Gastrointestinal Endoscopic Skills, VR – virtual reality.

**TABLE A2:** Teaching model‐type application.

Teaching method	Frequency of instances—*n* (%)	Frequency of studies—*n* (%)	Skill acquisition focus
Simulation			
VR model	16 (33.3%)	16 (52%)	Torque steering, loop reduction, tip control Anatomical orientation Complication recognition (bleed, perf, pressure management)
Physical model	13 (27.1%)	13 (42%)	Torque steering, loop reduction, tip control Anatomical orientation Mechanical feedback
Box model	5 (10.4%)	5 (16%)	Tip control Complication recognition (bleed, perf, pressure management)
Animal model	4 (8.3%)	4 (13%)	Nuanced scope manipulation and pressure control skills Realistic mechanical feedback Complication recognition (bleed, perf, pressure management)
Apprenticeship			
Traditional/verbal method	3 (6.3%)	3 (10%)	Nuanced scope manipulation and pressure control skills Patient‐specific anatomical orientation Clinician‐led error recognition and correction
Two‐person colonoscopy	1 (2.1%)	1 (3%)	Tip control Clinical communication Patient‐specific anatomical orientation
Cognitive			
Didactic model	2 (4.2%)	2 (6.55%)	Procedural knowledge Cognitive sequencing and decision‐making
Feedback			
Real‐time feedback devices	4 (8.3%)	4 (13%)	Loop reduction Mechanical feedback and pressure control Real‐time error recognition and correction

*Note:* The frequency of all models utilised totalled 48. The number of studies totalled 31.

Abbreviation: VR – virtual reality.
